# Phytochemical Profiling of *Ferula varia* Extract and Its Antibiofilm Activity Against *Streptococcus mutans*

**DOI:** 10.3390/molecules30214178

**Published:** 2025-10-24

**Authors:** Marlen K. Smagulov, Yana K. Levaya, Karakoz Zh. Badekova, Svetlana A. Ivasenko, Gayane A. Atazhanova, Vika Gabe, Margarita Yu. Ishmuratova, Tomas Kacergius

**Affiliations:** 1 Research Park of Biotechnology and Eco-Monitoring, Karaganda Buketov University, Universitetskaya Street, 28, Karaganda 100028, Kazakhstan; marlenkemel@mail.ru (M.K.S.); margarita.ishmur@mail.ru (M.Y.I.); 2 School of Pharmacy, Karaganda Medical University, Gogol Street, 40, Karaganda 100017, Kazakhstan; ivasenko-74@mail.ru (S.A.I.); g-atazhanova@mail.ru (G.A.A.); 3 Department of Physiology, Biochemistry, Microbiology and Laboratory Medicine, Institute of Biomedical Sciences, Faculty of Medicine, Vilnius University, M. K. Ciurlionio Str. 21, 03101 Vilnius, Lithuania; vika.gabe@mf.vu.lt (V.G.); tomas.kacergius@mf.vu.lt (T.K.)

**Keywords:** *Ferula varia*, biofilm inhibition, *Streptococcus mutans*, phytochemicals, antibacterial activity

## Abstract

Dental caries is a major global health concern, with *Streptococcus mutans* playing a key role in biofilm formation and acid production, which lead to enamel demineralization. Natural products, particularly plant-derived extracts, offer promising alternatives to conventional antibacterial agents. This study aimed to analyze the chemical composition of *Ferula varia* 70% ethanol extract (FVE) and evaluate its potential to inhibit biofilm formation by *S. mutans*. The aerial parts of *F. varia* were extracted with 70% ethanol and analyzed using LC-UV-ESI-MS/MS to determine the chemical profile. The anti-biofilm activity of FVE was evaluated using a crystal violet assay against *S. mutans*. Phytochemical analysis identified 14 compounds, including major phenolic acids (e.g., chlorogenic acid, gallic acid) and flavonoids (e.g., isoquercitrin, isorhamnetin-3-O-glucoside). FVE exhibited significant, dose-dependent inhibition of *S. mutans* biofilm formation. Importantly, the FVE concentration of 5 mg/mL inhibited *S. mutans* biofilm development by 100%. The potent antibiofilm activity of FVE against *S. mutans* is likely due to the synergistic action of its rich content of phenolic acids and flavonoids, which possess known anti-virulence properties. These findings support the potential use of FVE as a natural ingredient in oral hygiene products to prevent dental plaque and caries.

## 1. Introduction

According to a report by the World Health Organization for the first quarter of 2025, nearly 3.7 billion people worldwide suffer from oral diseases. In addition, about 2 billion people worldwide suffer from caries in their permanent teeth, and nearly 514 million children are affected by caries in their primary teeth [1]. Thus, the growing prevalence of oral diseases is expected to drive demand in the global market for dental infection control products.

Dental caries remains a widespread oral health problem globally, primarily driven by the formation of biofilms and acid production by cariogenic bacteria, particularly *Streptococcus mutans*. The persistent and structured nature of biofilms makes them highly resistant to conventional antibacterial treatments. As a result, there is growing interest in plant-derived compounds with biofilm-inhibitory properties for use in oral healthcare [2]. Among oral pathogens, *S. mutans* is considered the primary etiological agent of dental caries due to its ability to form stable biofilms on tooth surfaces [3]. Inhibiting *S. mutans* biofilm formation is thus a promising strategy for caries prevention and treatment.

Modern methods of combating dental biofilm include mechanical removal and the use of broad-spectrum non-specific antibiotics [4]. However, teeth cleaning needs to be carried out regularly because the biofilm builds up quickly, and the antibacterial ingredients in mouthwashes (e.g., chlorhexidine (CHX), delmopinol hydrochloride) act non-selectively, affecting both microbiota and potentially causing side effects such as nausea, diarrhea, tooth discoloration, and habituation [5]. Various biologically active compounds (BAC) such as flavonoids, phenolic acids, terpenoids, etc., exhibit antibacterial activity against *S. mutans* and inhibit biofilm formation by suppressing adhesion, exopolysaccharide synthesis, and virulence gene expression. Thus, thymol and sabinene disrupt membrane integrity and suppress key genes (*gtfB*, *gtfC*, *gtfD*), gallic acid and ethyl gallate reduce cell viability and glucan synthesis, and magnolol surpasses CHX in bactericidal activity in biofilms. CHX in toothpaste reduces the amount of *S. mutans* in vivo, ginkgolic and ent-trachylobanic acids inhibit growth and adhesion, and epipimaric and shikimic acids prevent exopolysaccharide (EPS) attachment and synthesis. Quercetin, kaempferol, myricetin, and procyanidin A2 also effectively reduce biofilm mass and virulence [2]. These data confirm the potential of certain plant metabolites as antibacterial agents for the prevention of dental caries. Plant extracts, including green tea, pomegranate, garlic, turmeric, and black walnut, have pronounced antibacterial activity against *S. mutans* and are capable of inhibiting biofilm formation. Green tea catechins reduce exopolysaccharide synthesis, pomegranate tannins prevent bacteria from adhering to enamel, turmeric inhibits polysaccharide production, and garlic allicin and black walnut compounds effectively destroy bacterial cells and prevent biofilm formation [2,6,7].

Natural products derived from medicinal plants are a rich source of BACs, and many of them have served as the basis for the development of new dental therapeutic and prophylactic agents. As for diseases caused by microorganisms, the growing resistance of many common pathogens to currently used therapeutic agents, such as antibiotics and antiviral drugs, has led to renewed interest in the discovery of new antibacterial plant-based remedies.

It is known that plant components have antibacterial properties, helping to reduce the number of *S*. *mutans* colonies and inhibiting the synthesis of EPS matrix extracts necessary for biofilm formation. For example, essential oils have shown high activity against *S. mutans* and their biofilms [8]. Thus, antimicrobial gels and rinses containing *Melaleuca alternifolia* essential oil reduced the number of pathogenic bacteria and the gingival bleeding index [9]. Nanogels with peppermint essential oil suppressed key glycosyltransferase genes, confirming the potential of essential oils in toothpastes and mouthwashes [10]. Clinical trials have demonstrated the effectiveness of mouthwashes containing essential oils (e.g., Listerine, PerioGard with chamomile essential oil)—their effect was comparable to or superior to CHX-based preparations [9]. Synergy between thyme essential oil and CHX was also identified, and combinations with xylitol enhanced the antibacterial effect [11,12]. With growing interest in natural products, essential oils, extracts, and essences from plants (mint, rosemary, citrus, etc.) are seen as a promising alternative to traditional chemical ingredients in oral hygiene products.

The genus *Ferula* (Apiaceae), comprising over 150 species distributed across Central Asia, Kazakhstan, Western Siberia, China, and Mongolia [13], is known for its rich phytochemical profile and diverse biological activities. In Kazakhstan alone, 48 species of *Ferula* are recorded, including 16 endemic species [14]. Plants of this genus contain various biologically active compounds, such as coumarins [15,16], mono- and sesquiterpenes [17], and flavonoids [18]. Several *Ferula* species have demonstrated notable antimicrobial and anticariogenic properties [19,20], including inhibition of *S. mutans* growth, reduction in acid production, and suppression of biofilm formation. For example, ethanol extracts of *F. assa-foetida* have shown inhibitory activity against *S. mutans* and *Lactobacillus acidophilus*, suggesting potential application in oral hygiene products. Similarly, essential oil from *F. gummosa* exhibited strong biofilm-inhibitory and antimicrobial effects against oral pathogens such as *S. aureus*, *E. faecalis*, *S. mitis* and *C. albicans* [21]. A comprehensive review by Salehi et al. [22] highlights the anti-inflammatory, antioxidant, and antimicrobial properties of various *Ferula* species, indicating their potential use in oral health. Boghrati et al. [23] reviewed the presence of bioactive compounds—such as coumarins and terpenoids—in *Ferula* spp. which are associated with antibacterial and anti-inflammatory effects.

In this context, *Ferula varia* is of particular interest—a little-studied but promising species with a rich phytochemical composition and high availability of raw materials in Kazakhstan and Central Asia. It is a perennial plant, 60–120 cm tall, widely distributed in Central Asia. It typically grows in fixed sandy soils, loess foothills, and areas with mixed vegetation. In traditional medicine, infusions of the plant’s roots are used to treat fever, toothache, and helminth infections, while the above-ground parts are applied externally for wound healing and internally for lactogenic effects [24]. All of these pharmacological effects are due to the rich content of BAC, including flavonoids, sesquiterpenes, coumarins, and polysulfides. There are 51 species of *Ferula* spp. growing in Kazakhstan, 15 of which are endemic, and another 15 are listed in the country’s Red Book of Flora, which highlights the uniqueness of local species and their potential for developing innovative herbal remedies. Phytochemical studies indicate that the aerial parts of *F. varia* are rich in flavonoids, predominantly luteolin and cynaroside [25]. Cynaroside is of particular pharmacological interest due to its hypoazotemic effects [26], and its content (~1.0%) has been proposed as a standardizing marker in quality control of *F. varia* raw materials.

It is known that low molecular weight metabolites of phenolic acids and flavonoids are found in most plants. The choice of *F. varia* was determined by a number of factors, namely the facts that the phytochemical profile of *F. varia* goes beyond the usual phenolic compounds and this species is widely distributed in Kazakhstan and Central Asia, which ensures the availability and replenishment of the raw material base, as well as the possibility of standardization and subsequent implementation into practice without dependence on imported raw materials. *F. varia* is rich in sesquiterpene lactones and coumarins, which are found in limited quantities in other species and have proven antimicrobial and antibacterial properties. In previous studies conducted by Suzuki et al. [14], six new sesquiterpene lactones and five known sesquiterpenes were isolated from *F. varia* roots, which complements the overall chemical characterization of the species and indicates its potential biological activity. The biologically active compounds of *F. varia* have a complex effect: they reduce adhesion and have antioxidant and anti-inflammatory properties, which helps to reduce both the number and the pathogenicity of bacteria [27]. These components are capable of inhibiting the growth of *S. mutans*, suppressing acid production, and reducing the formation of biofilm, which plays a key role in the development of caries. This combination of phenolic compounds with unique secondary metabolites makes the extract multi-component and potentially more active.

This study profiles the phenolic components of *F. varia* extract for the first time and evaluates its ability to inhibit biofilm formation. *F. varia* has traditionally been used in Central Asia as a spice and medicinal plant. In folk medicine, resins and extracts of *Ferula* have been used for inflammatory diseases of the respiratory tract, infections, and as antiseptics. These data indicate that *Ferula* are generally considered plants with antibacterial potential, which creates the prerequisites for their use in dentistry. The BACs contained in *F. varia* have pronounced antibacterial, antioxidant, and anti-inflammatory properties, which makes them promising for inhibiting biofilms and preventing caries. Although the *Ferula* genus has been well studied in terms of ethno pharmacology and antibacterial activity, *F. varia* specifically remains understudied in dentistry. This study aims to investigate the effects of FVE on biofilm formation and viability of *S. mutans*, a key cariogenic bacterium involved in dental plaque formation. The findings may contribute to the development of novel strategies for caries prevention.

## 2. Results

### 2.1. Chemical Composition

The total phenolic content (TPC) of FVE was calculated from the regression equation of the calibration curve (*y* = 0.0382x + 0.0594, R^2^ = 0.993) and expressed as milligrams of gallic acid equivalents per gram of dry extract (mg GAE/g) per gram. The TPC was determined to be 23.22 ± 1.02 mg GAE/g. The total flavonoid content (TFC) was calculated from the calibration plot (*y =* 0.0376x + 0.018, *R*^2^ = 0.997) and expressed as mg of milligrams of rutin equivalents per gram of dry extract (mg RUE/g) per gram. The TFC was found to be 2.83 ± 0.98 mg RUE/g dry extract.

**Figure 1:**
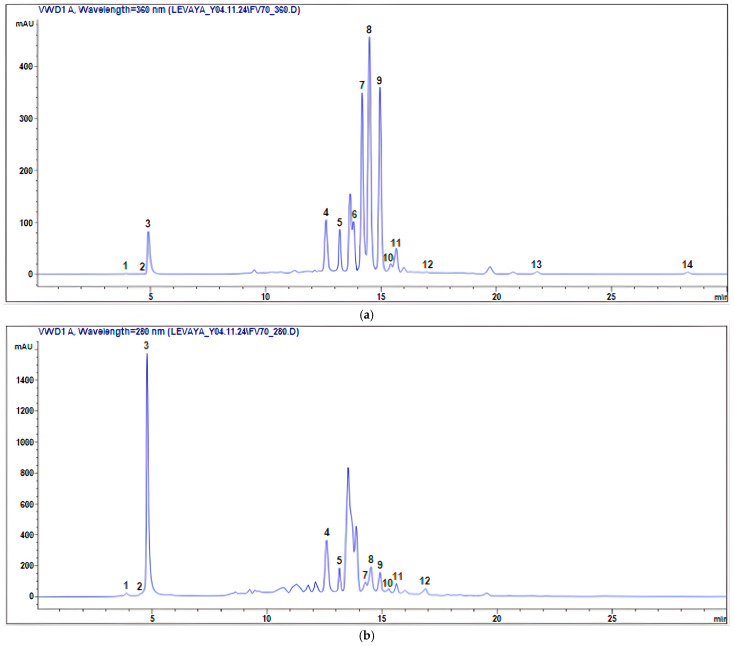
HPLC-UV chromatograms of *F. varia* 70% ethanol extract: (**a**) 360 nm, (**b**) 280 nm.

The results for polyphenols determination using LC-UV-ESI-MS/MS in the FVE are given in Table 1 and Table S1, chromatograms are presented in Figure 1, Figures S1 and S2.

**Figure 2:**
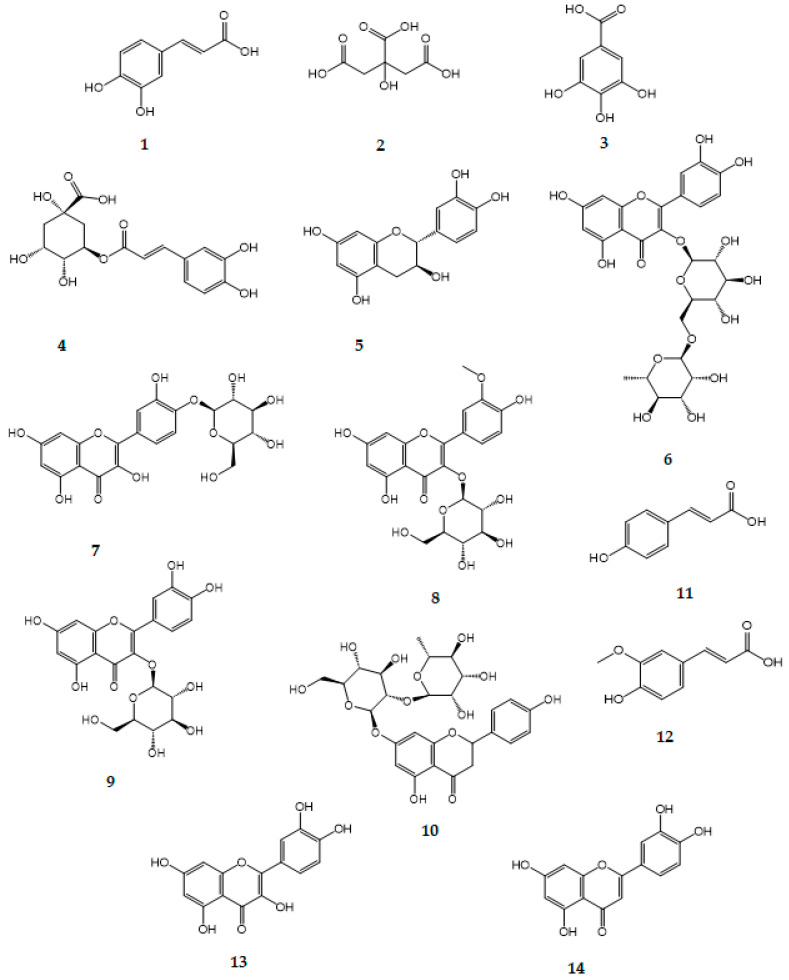
Phenolic compounds detected using HPLC-UV in *F. varia* 70% ethanol extract.

A total of 14 polyphenols were identified (Figure 2). The most abundant compound detected was chlorogenic acid **4**, with a concentration of 69.45 ± 1.12 mg/g extract, followed by gallic acid **3** at 24.01 ± 0.36 mg/g extract, and isoquercitrin **9** at 10.59 ± 0.30 mg/g extract. Other notable phenolic constituents included isorhamnetin-3-*O*-*β*-D-glucopyranoside **8** (9.05 ± 0.32 mg/g), spiraeoside **7** (5.72 ± 0.24 mg/g), and catechin **5** (3.63 ± 0.16 mg/g). Minor components such as quercetin **13** (0.19 ± 0.16 mg/g) and luteolin **14** (0.09 ± 0.04 mg/g) were also detected in lower amounts.

Phenolic acids including caffeic **1**, citric **2**, ferulic **12**, and p-coumaric **11** acids were also present in moderate concentrations ranging from 1.12 to 1.98 mg/g.

Results of TPC, TFC and HPLC revealed that *F. varia* contains a relatively high amount of phenolic acids compared to flavonoids, indicating that phenolic compounds may play a more prominent role in potential pharmacological activities.

### 2.2. Antibacterial Activity of Ferula varia 70% Ethanol Extract on S. mutans

As shown in Table 2, the value of the MIC for the effect of FVE on *S. mutans* is 25 mg/mL.

### 2.3. Biofilm Inhibition

**Figure 3:**
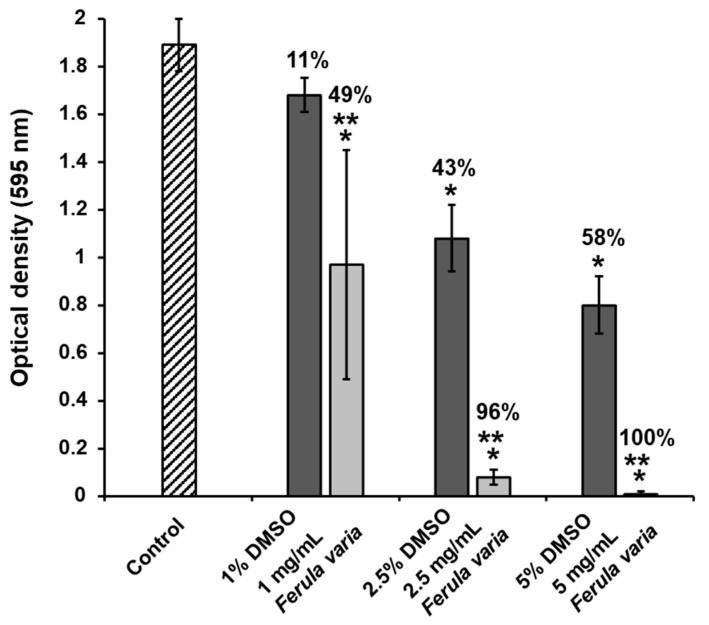
Levels of *Streptococcus mutans* biofilm following 24 h of exposure to various concentrations of *Ferula varia* 70% ethanol extract, produced with assistance of ultrasound, and dimethyl sulfoxide (DMSO). Data are presented as the mean ± standard error from four independent experiments (*n* = 4–18). * *p* < 0.05 compared to the control (untreated bacteria); ** *p* < 0.05 compared to the DMSO.

Exposure of *S. mutans* bacteria to FVE, produced with assistance of ultrasound, resulted in a great reduction in the biofilm biomass on polystyrene surfaces. As demonstrated in Figure 3, the FVE concentrations of 1, 2.5 and 5 mg/mL diminished *S. mutans* biofilm development in a dose-dependent manner by 49, 96 and 100%, respectively. It should be highlighted that the antibiofilm activity of FVE was significant versus the control and DMSO groups (*p* < 0.05).

## 3. Discussion

### 3.1. Chemical Characterization of Ferula varia 70% Ethanol Extract

One of the main objectives of the present study was to perform a comprehensive chemical characterization of the FVE. Hence, LC-UV-ESI-MS/MS analysis was performed and revealed the presence of 14 constituents, including 8 flavonoids, 5 phenolic acids and 1 organic acid presented by citric acid. Chlorogenic **4** 69.45 ± 1.12 mg/g and gallic **3** acids 24.01 ± 0.36 mg/g, isoquercitrin **9** 10.59 ± 0.30 mg/g, isorhamnetin-3-*O*-*β*-D-glucopyranoside **8** 9.05 ± 0.32 mg/g, and spiraeoside **7** 5.72 ± 0.24 mg/g identified, the presence of which could contribute to the observed TPC and TFC values. Phenolic acids are characterized by the presence of an aromatic ring with hydroxyl and carboxyl groups, which determines their high antioxidant activity. Chlorogenic acid **4** may also participate in antibacterial and hepatoprotective activity [38]. The highest content among flavonoids was observed in isoquercitrin **9** and isorhamnetin-3-*O*-*β*-D-glucopyranoside **8**, both of which are glycosides of quercetin and have pronounced antioxidant, anti-inflammatory, and capillary-strengthening properties [39]. The presence of both aglycones (e.g., quercetin, luteolin) and glycosides indicates the diversity of the flavonoid profile of *F. varia*, which may be related to its adaptation to stressful environmental conditions. The TPC and TFC observed in *F. varia* compared to other *Ferula* species may be attributed to a combination of genetic, ecological, and environmental factors. *F. varia* is primarily distributed in arid and semi-arid regions of Central Asia, where exposure to environmental stressors such as intense solar radiation, high temperature fluctuations, and limited water availability may stimulate the biosynthesis of phenolic secondary metabolites as part of the plant’s defense mechanism. In contrast, other *Ferula* species adapted to more temperate or mesic environments may not experience the same level of abiotic stress, resulting in comparatively lower accumulation of these compounds. Moreover, interspecific differences in metabolic pathways, particularly in the expression of key enzymes involved in phenylpropanoid and flavonoid biosynthesis (e.g., PAL, CHS, FLS), may also contribute to the elevated phenolic and flavonoid profiles in *F. varia*. This phytochemical richness could underlie the stronger antioxidant and antimicrobial activity demonstrated by this species and supports its distinct chemotaxonomic position within the genus. Future comparative metabolomic and transcriptomic studies could help clarify these interspecific variations more precisely.

Since there is no data on *F. varia* chemical composition in the literature, we will compare the chemical composition with other types of *Ferula*. Authors [28] reported a comprehensive chemical characterization of *F. persica* extract obtained with PLE, tentatively identified 170 compounds represented hydroxycinnamic acids and derivatives, benzoic acids and derivatives, and flavonoids. The predominance of phenolic compounds in *F. communis* fruit extract have been confirmed using HPLC-DAD analysis by Nouiuura et al. [18]. Leaf extract GC-MS profile of *F. asafetida* constituted high levels of carvacrol (15.40%) and α-bisabolol (9.75%), while phenolic profile by HPLC analysis constituted presence of ferulic acid, vanillic acid, coumaric acid, umbelliferone, galbanic acid, karatavicinol, and kamolonol which may contribute to antimicrobial and antioxidant activity [32]. Essential oils analysis of *F. latisecta* aerial parts using GC/MS revealed presence of (*Z*)-Ocimenone (32.4%), (*E*)-ocimenone (20.3%), and *cis*-pinocarvone (11.4%) [40]. According to a number of studies, species of the genus *Ferula* are characterized by a high content of sesquiterpene coumarins, ferulic resins, and essential oils rich in monoterpenes and sesquiterpenes [41,42].

Obtained results of TPC and TFC significantly lower than reported by other research and was determined as 23.22 ± 1.02 mg GAE/g DW and 2.83 ± 0.98 mg RUE/g DW. It was found that ethanol extract of *F. communis* exhibited the highest yielding TPC 62.20  ±  0.11 mg GAE/g DW and TFC 17.09 mg QE/g DW [18]. Similarly, Mohammadnezhad et al. [28], described TPC and TFC of aerial part and roots ethanol extracts of *F. persica* obtained by PLE under optimized conditions. TPC for aerial part and roots was found in amount of 113.5 ± 3.5 and 126.2 ± 3.9 mg GAE/g, TFC—16.0 ± 0. 4 and 3.0 ± 0.2 mg QE/g, respectively. Authors [43] reported TPC of 70% ethanol extract of 3 *Ferula* species growing in Tajikistan: *F. violacea*, *F. gigantean*, *F. kuhistanica* ranged from 990.7 up to 2176 expressed as μg GAE g^−1^. In general, the observed differences in the TPC and TFC of *Ferula* species can be influenced by multiple factors, including the specific species or variety studied, the plant part used (e.g., roots, aerial parts, oleo-gum-resin), and the extraction method applied. Key methodological parameters such as the type of solvent (e.g., methanol, ethanol, water), extraction temperature, duration, and solvent-to-sample ratio play a critical role in the efficiency of phenolic compounds recovery. In addition, environmental factors like geographical origin, soil composition, and harvesting season may significantly affect the phenolic composition. It is also important to note that TPC and TFC values may vary depending on the calibration standard used for quantification directly impacts the expression of results. Therefore, comparisons between studies should be made with caution, considering all methodological and analytical differences.

### 3.2. Inhibition of Streptococcus mutans Biofilm Formation

This study demonstrates that the FVE, obtained via ultrasound-assisted extraction, exhibits a strong, dose-dependent antibiofilm activity against *S. mutans*. It is important to note that the biofilm inhibiting FVE concentrations of 1, 2.5, and 5 mg/mL are lower than the determined minimum inhibitory concentration MIC (i.e., 25 mg/mL). Therefore, this indicates that the inhibitory activity was not related to the bactericidal effect of FVE. Although FVE achieved 100% inhibition of *S. mutans* biofilm formation at 5 mg/mL, this concentration is considerably higher than those reported for individual compounds (50–200 μg/mL), and therefore a synergistic effect cannot be concluded. The moderate inhibition rate (49%) observed at 1 mg/mL may reflect concentration-dependent, non-linear responses or potential antagonistic interactions among constituents within the crude extract. These results highlight the potential of *F. varia* as a natural source of antibiofilm agents for oral health applications.

The strong inhibitory effect may be attributed to the diverse composition of the FVE, which likely includes a mixture of phenolic acids, flavonoids, and flavonoid glycosides—several of which have been previously reported to inhibit *S. mutans* biofilm formation [44,45]. Niu et al. [46] reported that caffeic acid has been shown to reduce adherence and inhibit glucosyltransferases (*Gtfs*), which are essential for EPS production and biofilm integrity in *S. mutans*. Considering that chlorogenic and gallic acids are one of the predominant components identified in the *F. varia* extract, it is reasonable to assume that it plays a significant role in inhibiting *S. mutans* biofilm formation. In the virtual screening conducted by Liu et al. [47] chlorogenic acid from *Lonicera japonica flos* was found to be a potential inhibitor of *S. mutans* biofilm and had a wider inhibitory concentration range (3.0–0.0938 mg/mL) in a concentration-dependent way. Gallic acid demonstrated a notable inhibitory effect on *S. mutans* biofilm formation, with its efficacy influenced by environmental factors such as temperature, nutrient availability, and exposure time. In the study by Shao et al. [48], gallic acid showed inhibitory activity against *S. mutans* biofilm formation, with a minimum inhibitory concentration of 8 mg/mL. Quercetin showed significant antibiofilm activity against *S. mutans*, reducing biofilm biomass, protein content, viable cell counts, and glucan production, while also maintaining a less acidic environment [49]. The study showed that flavonoids such as isorhamnetin and rutin have a MIC above 500 μM against *S. mutans*, indicating a limited antibacterial effect of this group of compounds [50]. The results from a previous study [51] have shown that luteolin strongly inhibit *S. mutans* biofilm formation by reducing bacterial biomass, extracellular polysaccharide production, and acidogenicity. It downregulated virulence genes (*gbpC*, *spaP*, *gtfBCD*, *ftf*) and interfered with glucosyltransferases and amyloid proteins, demonstrating potent anti-biofilm activity without affecting bacterial viability.

Notably, many of the individual compounds have demonstrated moderate inhibitory effects on *S. mutans* biofilm formation typically 40–70% inhibition at concentrations between 50–200 µg/m). The FVE, however, achieved complete inhibition 100% at a higher concentration of 5 mg/mL. Given the difference in concentration ranges tested, a direct comparison between the extract and isolated compounds is not appropriate, and no conclusion regarding synergistic effects can be drawn at this stage. Nevertheless, the potent antibiofilm activity of FVE highlights its potential as a source of antimicrobial agents for developing effective plant-based caries prevention products.

## 4. Materials and Methods

### 4.1. Plant Material

The wild plant *Ferula varia* (Schrenk ex Fisch., C.A.Mey. &Avé-Lall.) Trautv. of the flora of Kazakhstan was collected during the flowering phase in May 2023 during expeditions to the Pribalkhash region (Republic of Kazakhstan). The collection was made in the vicinity of the Bektatau Mountains, Aktogai District, Karaganda Region, collection coordinates (47.74108 N, 74.295 E). Coarse stems are removed from the dry raw material of *F. varia*. Flower baskets and leaves are crushed to 2–3 mm. The above-ground parts of *F. varia* were verified by botanist M. Yu. Ishmuratova and placed in the herbarium of the E.A. Buketov Karaganda University under number QAR04064.

### 4.2. Chemicals and Solvents

Solvents for LC-UV-ESI-MS/MS analysis: acetonitrile (ACN) for HPLC (≥99.9%, Sigma-Aldrich, Saint-Quentin-Fallavier, France), formic acid (99–100%, AnalaR NORMAPUR^®^, VWR Chemicals, Rosny-sous-Bois, France), highly purified water was prepared using a Milli-Q water purification system (Millipore, Molsheim, France). Gallic acid, caffeic acid, citric acid, chlorogenic acid, ferulic acid, p-coumaric acid, catechin, naringin, rutin, spiraeoside, isorhamnetin-3-*O*-*β*-D-glucopyranoside, isoquercitrin, quercetin, luteolin, ethanol, Folin–Ciocalteu reagent, sodium carbonate, sodium nitrite, aluminum chloride, sodium hydroxide were purchased from Sigma–Aldrich (Saint Louis, MO, USA).

### 4.3. Preparation of the Ferula varia 70% Ethanol Extract (FVE)

50.0 g of *F. varia* herb is placed in a container for extracting raw materials, then poured with a pre-prepared extractant—70% ethanol, with a ratio of raw material mass to extractant volume of 1:10 (g/mL). Ultrasonic extraction of *F. varia* raw materials is carried out without soaking on an ultrasonic cleaner at an ultrasonic radiation frequency of 40 kHz, at room temperature (20–22 °C), for 30 min [52]. Then, the liquid extract is drained and the extraction of the raw material is repeated once more under the same conditions. The combined liquid extract of *F. varia* is filtered through a paper filter. The extractant is evaporated in a rotary evaporator RV8 (IKA Gmbh, Staufen, Germany) at a temperature of 50 °C under vacuum. The residual solvent is evaporated from the thick extract in a water bath at a temperature of 70 °C, giving brownish dry extract with yield 24.9%.

### 4.4. Determination of Total Phenolic Content

TPC of the FVE was determined using the Folin–Ciocalteu colorimetric method, as originally described by Singleton and Rossi, with minor modifications [53]. Standard solutions of gallic acid were prepared in the concentration range of 100–500 µg/mL.

For sample analysis, the extract was dissolved in ethanol. Then, 100 µL of this extract solution (or gallic acid standard) was mixed with 500 µL of diluted Folin–Ciocalteu reagent (1:10 *v*/*v* in distilled water) and incubated for 6 min at room temperature. Subsequently, 400 µL of sodium carbonate solution (Na_2_CO_3_) (7.5% *w*/*v*) was added, and the mixture was incubated for 15 min at 40 °C. Absorbance was then measured at 765 nm using a UV-Vis spectrophotometer Cary 60 UV-Vis (Agilent, Bayan Lepas, Penang, Malaysia).

The phenolic content was quantified using the gallic acid calibration curve, and concentrations were calculated from the regression equation derived from the standard absorbance values. All measurements were performed in triplicate, and results were expressed mg GAE/g of dry extract using this equation [53].

C = C_1_ × V/m

where C is TPC in mg/g, in GAE, C_1_ is the concentration of gallic acid in μg/mL, obtained from the calibration curve, V is the sample volume in mL, m is the weight of the dry extract in mg.

### 4.5. Determination of Total Flavonoid Content

TFC of the FVE was determined using aluminum chloride colorimetric method based on previously reported procedures [54]. For total flavonoid determination, rutin was used to make the standard calibration curve. Standard solutions of rutin were prepared in the concentration range of 10–100 µg/mL.

For the sample preparation, 20 mg of the *F. varia* extract was dissolved in 10 mL of ethanol. Then, 1 mL of this solution was mixed with 4 mL of distilled water and 0.3 mL of 5% sodium nitrite (NaNO_2_) solution. After 5 min of incubation, 0.3 mL of 10% aluminum chloride (AlCl_3_) solution was added, followed by a 6 min incubation. Subsequently, 2 mL of 1 M sodium hydroxide (NaOH) solution was added to the mixture. The absorbance was measured at 415 nm using a UV-Vis spectrophotometer.

The concentration of flavonoids in the extract was calculated from the calibration curve using the regression equation obtained from the rutin standards. All measurements were performed in triplicate, and results were expressed as milligrams of rutin equivalents per gram of dry extract (mg RUE/g).

C = C_1_ × V/m

where C is TFC in mg/g, in RUE, C_1_ is the concentration of rutin in μg/mL obtained from the calibration curve, V is the sample volume in ml, m is the weight of the dry extract in mg.

### 4.6. LC-UV-ESI-MS/MS Analysis

The analysis was performed using an Agilent 1260 Infinity HPLC instrument (Agilent Technologies, Santa Clara, CA, USA) equipped with a G1314C 1260 VWD VL+ variable wavelength UV detector and a G6130A LC-MS/MS quadrupole mass spectrometer (Agilent Technologies, Santa Clara, CA, USA), an Eclipse Pluse C18 column (150 mm × 4.6 mm, 3.5 µm, Agilent Technologies, USA) according to the method [55]. Gradient elution was used for separation in a solvent system: A (2.5% formic acid in water (*v*/v)) and B (2.5% formic acid in acetonitrile (*v*/*v*)). Gradient elution program: 0.00 min—3% B, 7.00 min—20% B, 7.10 min—30% B, 27.00 min—40% B, 35.00 min—50% B, 35.10 min—20% B, and 40.00 min—3% B. The flow rate was 0.4 mL/min, the column temperature was 30 °C, and the sample volume was 20 μL. The separation process was monitored using a UV detector at 280 nm and 360 nm. Detection on the G6130A LC-MS/MS mass spectrometer was performed in negative mode with the following optimized parameters: capillary temperature 350 °C; drying gas N2–8 L/min; spray pressure 45 psi. The data were collected and processed using ChemStation B. 0403 SP1software based on Windows NT (Agilent Technologies, USA). Each compound was identified by comparing the obtained mass spectra with the mass spectra of standard samples and data from scientific literature.

### 4.7. Bacterial Strain and Culture Conditions

*Streptococcus mutans* strain UA159 (ATCC 700610) was cultured in Todd Hewitt broth (THB) under anaerobic conditions (95% N_2_ and 5% CO_2_) at 37 °C for 18 h prior to the experiments.

### 4.8. Microdilution Test for Determining the Minimum Inhibitory Concentration (MIC)

The broth microdilution assay was performed using two-fold serial dilution in Brain Heart Infusion (BHI) broth (Oxoid, UK). The test was carried out in 96-well flat-bottomed microtitration plates (Falcon, Corning Incorporated, Corning, NY, USA). The cell suspension was prepared in BHI broth with an optical density (OD) equivalent to 0.5 McFarland standard. The OD of prepared culture was measured using a microplate reader (Synergy HTX, BioTek Instrument Inc., Winooski, VT, USA). The absorption index was 0.08–0.1 at a wavelength of 600 nm. Prepared culture was diluted 1:100 in BHI broth to obtain a final concentration of 5 × 10^5^ colony forming units per milliliter (CFU/mL). Controls with broth only and broth with microbial cultures without any treatments were also included in the plates. 100 µL of the extract was put in the first microplate well and serially diluted in BHI broth. 100 µL, corresponding to 5 × 10^5^ CFU/mL, was added in all wells. The plates were incubated at 37 °C in 95% N_2_ and 5% CO_2_ for 18 h overnight. Two-fold dilution of chlorhexidine digluconate was used as positive control. The MIC was defined as the lowest concentration able to inhibit visible growth of microorganisms in triplicate wells. After visually determination of the MIC, 20 µL of Presto Blue reagent (Invitrogen, Carlsbad, CA, USA) was added to each well. The plates were incubated for 20 min, and then for 2 h, and afterwards evaluated visually for any change in color from blue to pink indicating reduction of dye due to microbial growth.

### 4.9. Biofilm Formation Assay

The FVE was screened for biofilm formation using methodology described Kacergius et al. [56]. 1000 mg of *F. varia* 70% ethanolic extract was dissolved in 10 mL of pure dimethyl sulfoxide in order to produce stock solutions with concentration of 100 mg/mL. The prepared stock solutions were stored at −35 °C until use.

At the beginning of experiments, 24-well, flat-bottomed, polystyrene cell culture plates were filled with the THB containing 1% sucrose, and then extract was added to the appropriate wells at final concentrations of 1 mg/mL, 2.5 mg/mL, 5 mg/mL, 7.5 mg/mL and 10 mg/mL. From this range, three concentrations of the plant extract were selected for the treatment of *S. mutans* bacteria. The solvent DMSO was added to the appropriate wells at final concentrations of 1%, 2.5%, 5%, 7.5% and 10% (*v*/*v*).

Prior to each experiment, the optical density of the bacterial culture was adjusted to 0.2 at 600 nm using a microplate-reader spectrophotometer Synergy HTX (BioTek Instrument Inc., USA). *S. mutans* bacteria were then added to the plate wells, containing the plant extracts, at a final dilution of 1:100, and all of the plates were incubated anaerobically (95% N_2_ and 5% CO_2_) at 37 °C for 24 h.

In the experiments, plate wells without bacterial cells were used as blank controls, whereas the untreated bacteria without sucrose served only as internal controls for the experiments and were not included in the calculations.

After 24 h of incubation, THB was discarded from the plates, the wells were rinsed with distilled water to remove loosely bound cells, and then adherent bacteria were fixed with 95% ethanol. The fixed and air-dried *S. mutans* biofilm in the plate wells was stained with 0.01% crystal violet solution for 15 min.

The bound dye was extracted using 33% acetic acid solution for 30 min. Afterwards, 200 µL of the extracted dye solution from each well was transferred to the appropriate wells in an optically clear, flat-bottomed, 96-well microplate. The OD of the extract was measured at a wavelength of 595 nm with a microplate-reader spectrophotometer Synergy HTX (BioTek Instrument Inc., USA). Background staining was corrected for by subtracting the amount of the staining in the blank wells.

The percentage of the inhibition of the biofilm formation was calculated using the values of OD according to the equation:

$$\%\;of\;the\;inhibition=\frac{{Parameter}_{control}-{Parameter}_{treatment}}{{Parameter}_{control}}\;\times 100\%$$

### 4.10. Statistical Analysis

The data were analyzed using SPSS version 23.0 (IBM Corp., Armonk, NY, USA). The differences between the control (untreated) and treatment groups were evaluated by applying a one-way analysis of variance, followed by a post hoc least significant difference test for multiple comparisons. The data are presented as the mean ± standard error (SEM). A *p* value of less than 0.05 was considered to indicate a statistically significant difference.

## 5. Conclusions

The comprehensive phytochemical characterization of the FVE aerial parts revealed a diverse profile of BAC, primarily phenolic acids and flavonoids, with chlorogenic acid (69.45 ± 1.12 mg/g) and gallic acid (24.01 ± 0.36 mg/g) identified as the major constituents. In total, fourteen phenolic compounds were identified by LC-UV-ESI-MS/MS, including both aglycones and glycosylated flavonoids such as isoquercitrin, isorhamnetin-3-*O*-glucoside, and spiraeoside. This chemical complexity likely underpins the observed biological activities and reflects the plant’s potential adaptation to environmental stressors.

FVE exhibited a significant dose-dependent inhibition of *S. mutans* biofilm formation, achieving complete eradication of biofilm biomass at a concentration of 5 mg/mL. Notably, these antibiofilm effects occurred at concentrations well below the determined MIC of 25 mg/mL, suggesting that the mechanism of action is not bactericidal but rather involves interference with biofilm development and virulence factors. This finding underscores the extract’s potential application in oral health, particularly in the prevention of dental caries. The presence of known antibiofilm agents—such as chlorogenic acid, gallic acid, quercetin, and luteolin—suggests that the strong bioactivity may result from synergistic or additive effects among the extract’s constituents. Such interactions are commonly observed in multi-component phytochemical preparations and often surpass the efficacy of isolated compounds.

In conclusion, *F. varia* demonstrates considerable promise as a natural source of phenolic compounds with potent antibiofilm activity against *S. mutans*. These results support the potential development of plant-based oral care products aimed at biofilm control and caries prevention. However, further investigations are warranted to elucidate the specific mechanisms of action, assess cytotoxicity and bioavailability, and validate these findings in in vivo and clinical settings.

## Figures and Tables

**Table 1:** Identification and content of phenolic compounds in *F. varia* 70% ethanol extract.

Peak/№	RT	M − H^−^	Compound Name	Quantitative Content (mg/g Extract)	Reference
1	3.878	179	Caffeic acid	1.98 ± 0.14	[18,28,29]
2	4.820	191	Citric acid	1.42 ± 0.12	[18,30,31]
3	4.944	169	Gallic acid	24.01 ± 0.36	[30,32,33]
4	12.671	353	Chlorogenic acid	69.45 ± 1.12	[8,30,31,33,34]
5	13.325	289	Catechin	3.63 ± 0.16	[18,33,35]
6	13.962	609	Rutin (Quercetin-3-*O*-rutinoside)	1.92 ± 0.16	[18,28,29]
7	14.262	463	Spiraeoside (Quercetin-4′-*O*-*β*-D-glucopyranoside)	5.72 ± 0.24	[35]
8	14.566	477	Isorhamnetin-3-*O*-*β*-D-glucopyranoside	9.05 ± 0.32	[35]
9	14.967	463	Isoquercitrin (Quercetin-3-*O*-*β*-D-glucopyranoside)	10.59 ± 0.30	[18,31]
10	15.293	579	Naringin (Naringenin-7-rhamnosidoglucoside)	0.29 ± 0.06	[18,36]
11	15.792	163	*p*-coumaric acid	1.40 ± 0.12	[18,30,32,34]
12	16.998	193	Ferulic acid	1.12 ± 0.14	[18,28,29,30,31,32,33,34]
13	21.894	301	Quercetin	0.19 ± 0.16	[28,31,33,36,37]
14	28.354	285	Luteolin	0.09 ± 0.04	[30,35]

**Table 2:** Antibacterial activity of *F. varia* 70% ethanol extract (stock solution 100 mg/mL dissolved in pure dimethyl sulfoxide (DMSO)), chlorhexidine digluconate (CHX, positive control), and DMSO (solvent).

Compound	MIC, mg/mL
*F. varia* extract	25 mg/mL
CHX	4.5 µg/mL
DMSO	50% (*v*/*v*)

## Data Availability

The original contributions presented in this study are included in the article/Supplementary Materials. Further inquiries can be directed to the corresponding authors.

## Supplementary Materials

The following supporting information can be downloaded at: https://www.mdpi.com/article/10.3390/molecules30214178/s1, Table S1. Identification of phenolic compounds in a 70% ethanol extract of F. varia based on HPLC-ESI-MS/MS analysis. Figure S1. HPLC-ESI-MS/MS total ion chromatogram (TIC) of F. varia 70% ethanol extract. Figure S2. HPLC-ESI-MS/MS selected ion chromatograms (SIM) of the identified phenolic compounds from F. varia 70% ethanol extract.
