# Supplementary material for: Phytochemical Profiling of Ferula varia Extract and Its Antibiofilm Activity Against Streptococcus mutans
Source: Molecules. 2025 Oct 24;30(21):4178. doi: 10.3390/molecules30214178 (PMC12610301; doi:10.3390/molecules30214178)
Supplement: Supplementary file 1 [file molecules-30-04178-s001.zip › molecules-3916251-supplementary.pdf]

**Table S1.** Identification of phenolic compounds in a 70% ethanol extract of F. varia based on HPLC-ESI-MS/MS analysis.

| Peak | Rt (min) | M–H⁻ (m/z) | Compounds | Molecular formula | Fragment | Reference |
|---|---|---|---|---|---|---|
| 1 | 3.878 | 179 | Caffeic acid | $C_9H_8O_4$ | 135, 107 | [8, 22, 23] |
| 2 | 4.820 | 191 | Citric acid | $C_6H_8O_7$ | 111, 87 | [8, 24, 25] |
| 3 | 4.944 | 169 | Gallic acid | $C_7H_6O_5$ | 125, 107, 79 | [24, 26, 27], |
| 4 | 12.671 | 353 | Chlorogenic acid | $C_{16}H_{18}O_9$ | 161, 143, 133 | [22, 24, 25, 27, 28] |
| 5 | 13.325 | 289 | Catechin | $C_{15}H_{14}O_6$ | 245, 205 | [8, 27, 29] |
| 6 | 13.962 | 609 | Rutin (Quercetin-3-*O*-rutinoside) | $C_{27}H_{30}O_{16}$ | 463, 301, 300 | [8, 22, 23] |
| 7 | 14.262 | 463 | Spiraeoside (Quercetin-4'-*O*-*β*-D-glucopyranoside) | $C_{21}H_{20}O_{12}$ | 301, 227, 151, 135 | [26] |
| 8 | 14.566 | 477 | Isorhamnetin-3-*O*-*β*-D-glucopyranoside | $C_{22}H_{22}O_{12}$ | 315, 300, 283, 151 | [26] |
| 9 | 14.967 | 463 | Isoquercitrin (Quercetin-3-*O*-*β*-D-glucopyranoside) | $C_{21}H_{20}O_{12}$ | 301, 271, 255, 179 | [8, 25] |
| 10 | 15.293 | 579 | Naringin (Naringenin-7-rhamnosidoglucoside) | $C_{27}H_{32}O_{14}$ | 459, 271, 151 | [8, 30] |
| 11 | 15.792 | 163 | *p*-Coumaric acid | $C_9H_8O_3$ | 119, 93 | [8, 24, 26, 28] |
| 12 | 16.998 | 193 | Ferulic acid | $C_{10}H_{10}O_4$ | 178, 149, 134 | [8, 22–28] |
| 13 | 21.894 | 301 | Quercetin | $C_{15}H_{10}O_7$ | 257, 179, 151 | [22, 25, 27, 30, 31] |
| 14 | 28.354 | 285 | Luteolin | $C_{15}H_{10}O_6$ | 217, 151, 133 | [24, 29] |

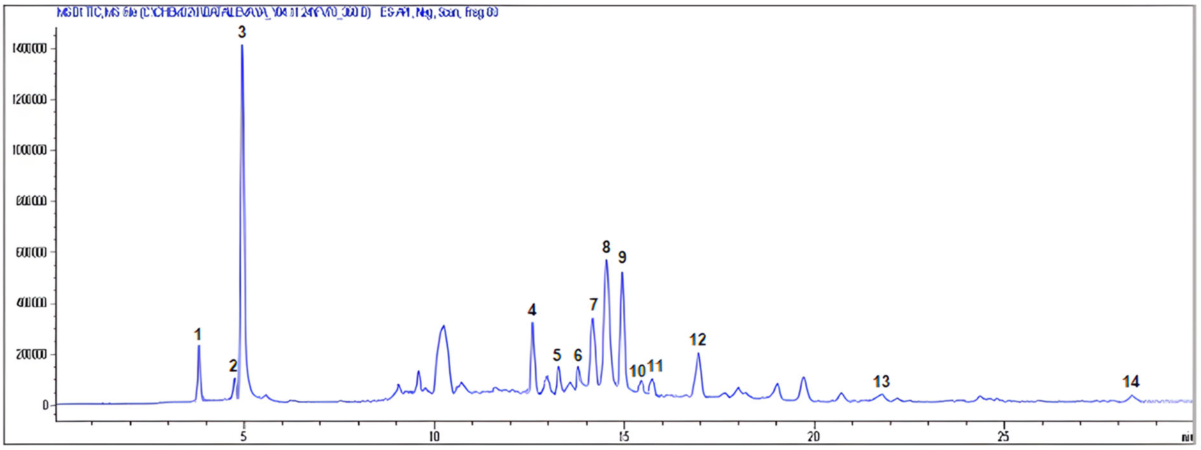


**Figure S1.** HPLC-ESI-MS/MS total ion chromatogram (TIC) of *F. varia* 70 % ethanol extract.

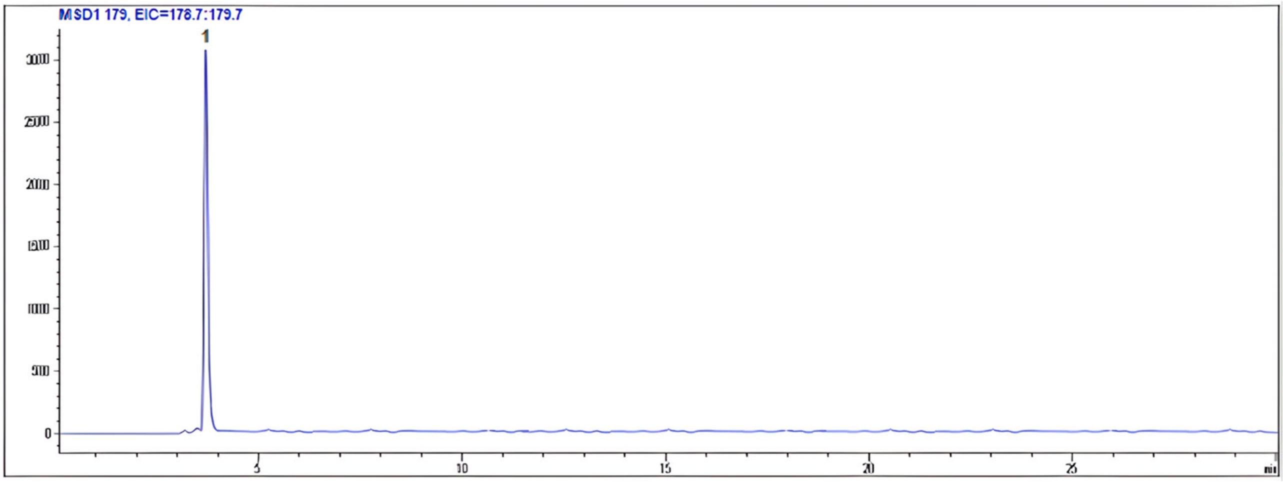


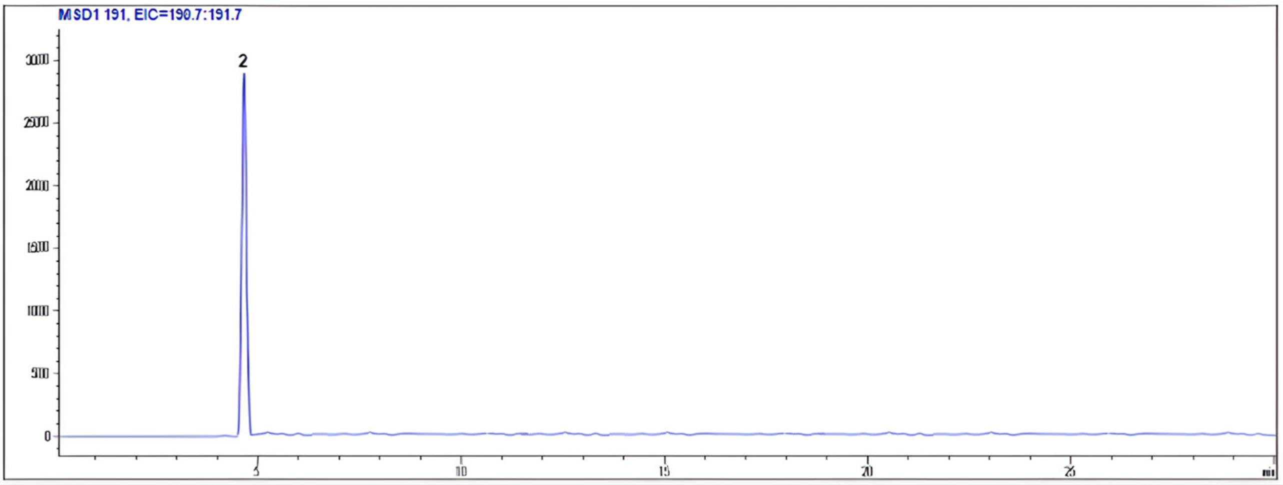

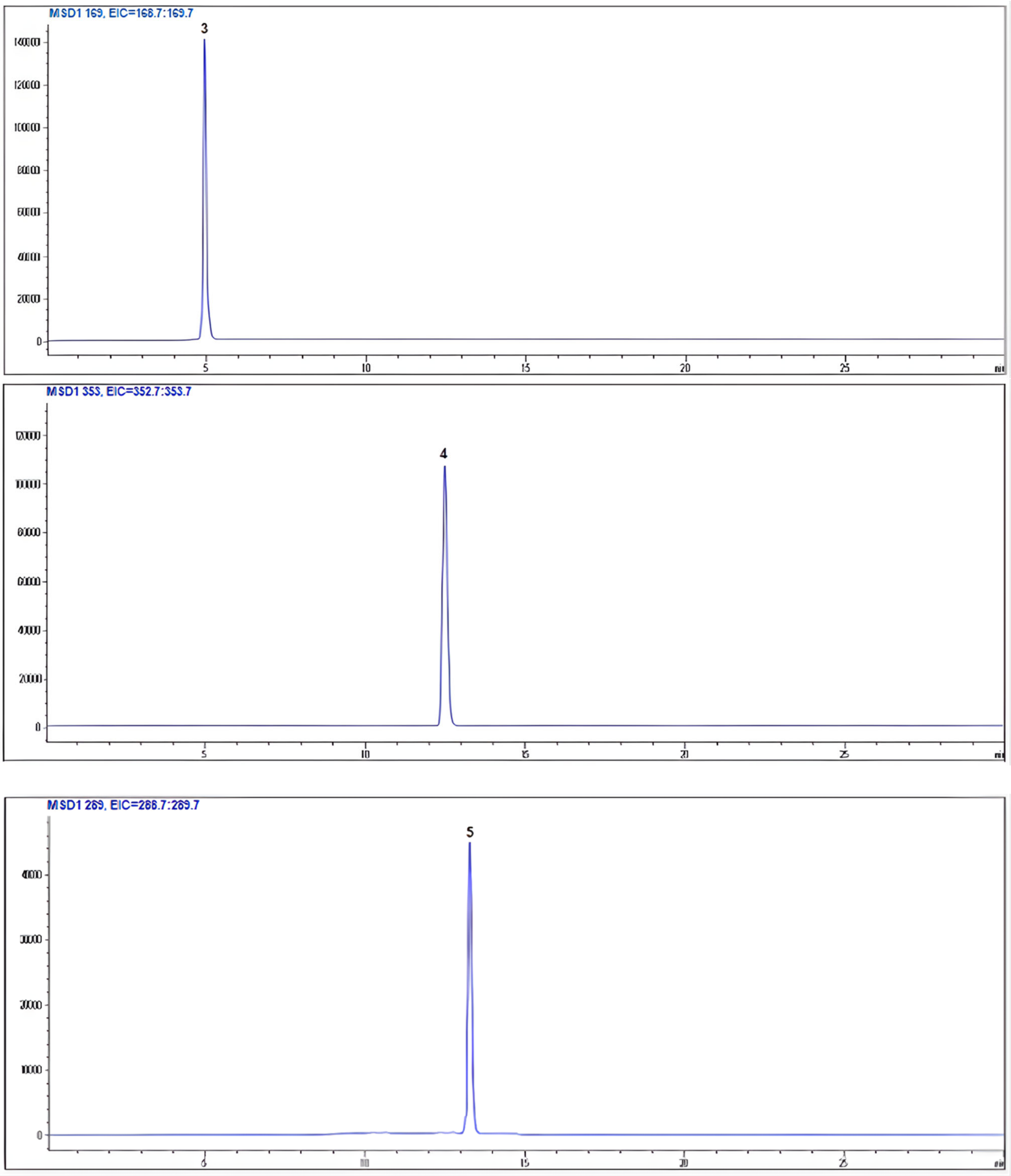
MSD1 169, EIC=168.7:169.7
3
140000
120000
100000
80000
60000
40000
20000
0
5
10
15
20
25
MSD1 353, EIC=352.7:353.7
4
120000
100000
80000
60000
40000
20000
0
5
10
MSD1 269, EIC=268.7:269.7
5
0
15
20

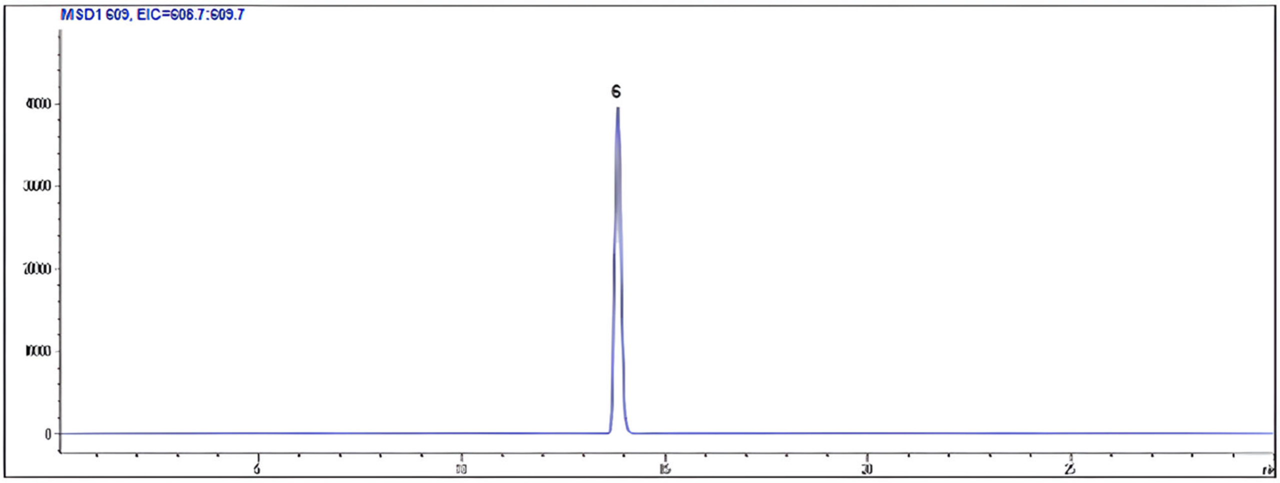
MSD1 609, EIC=608.7:609.7
6

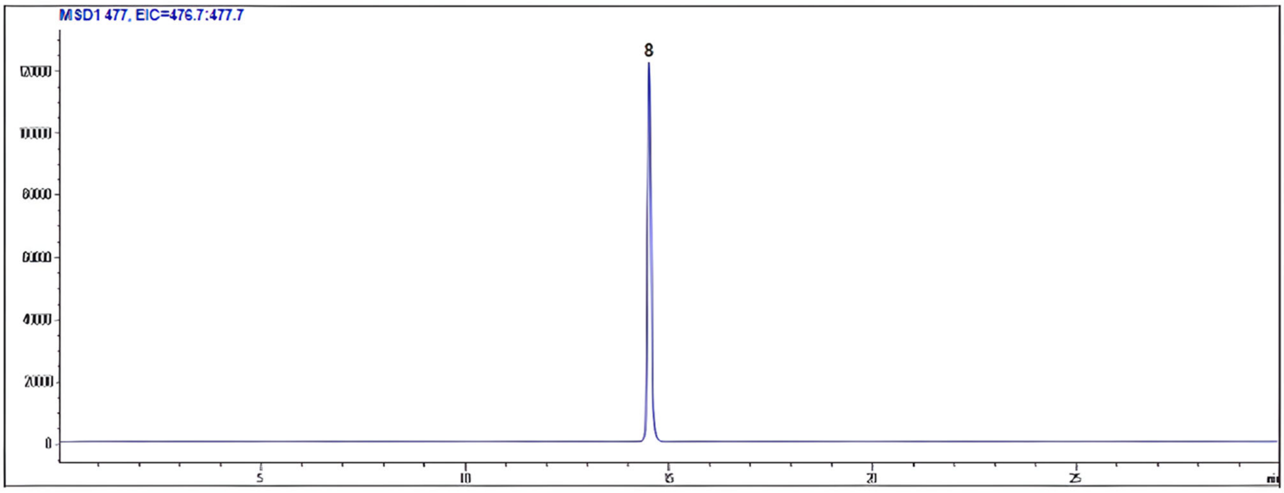
MSD1 477, EIC=476.7:477.7
8

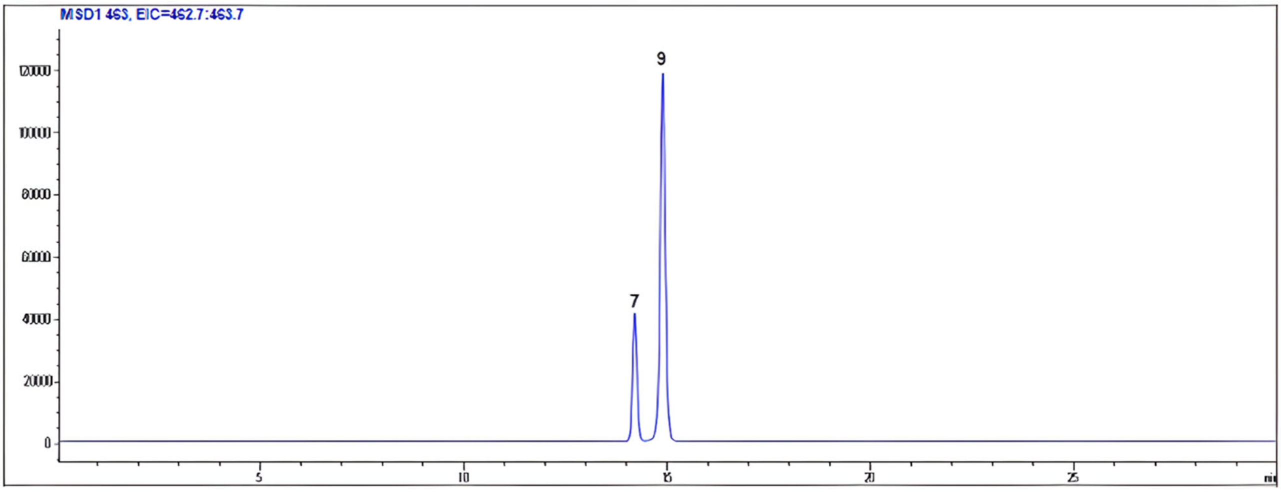
MSD1 463, EIC=462.7:463.7
9
7

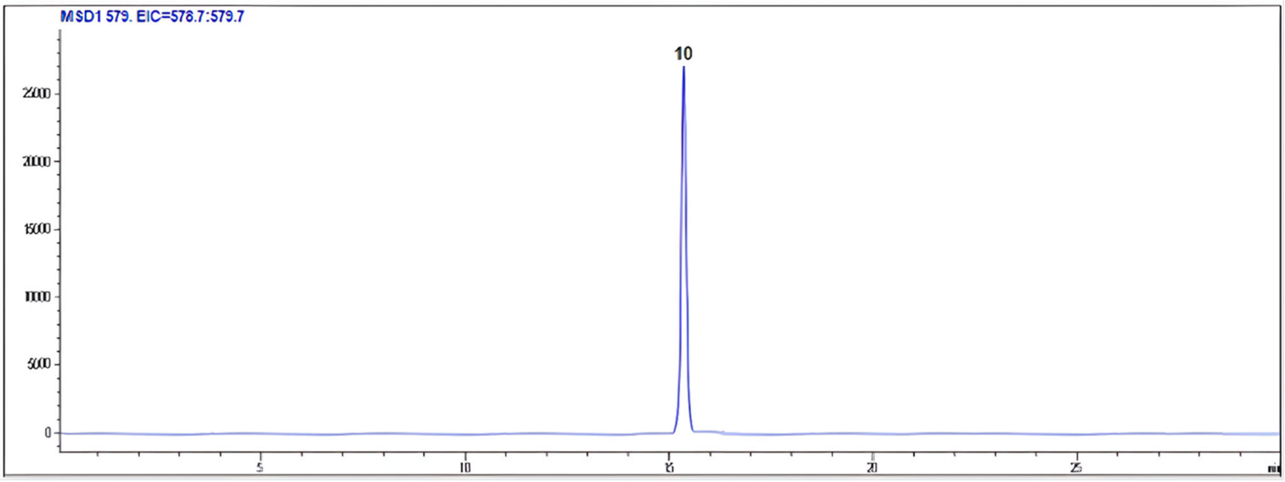
MSD1 579, EIC=578.7:579.7
10

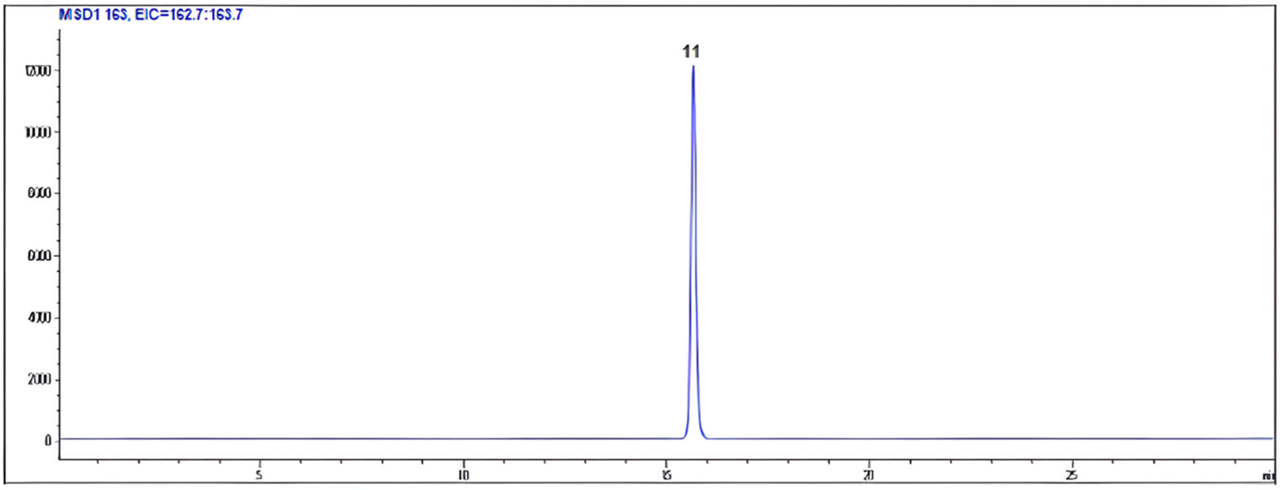
MSD1 163, EIC=162.7:163.7
11

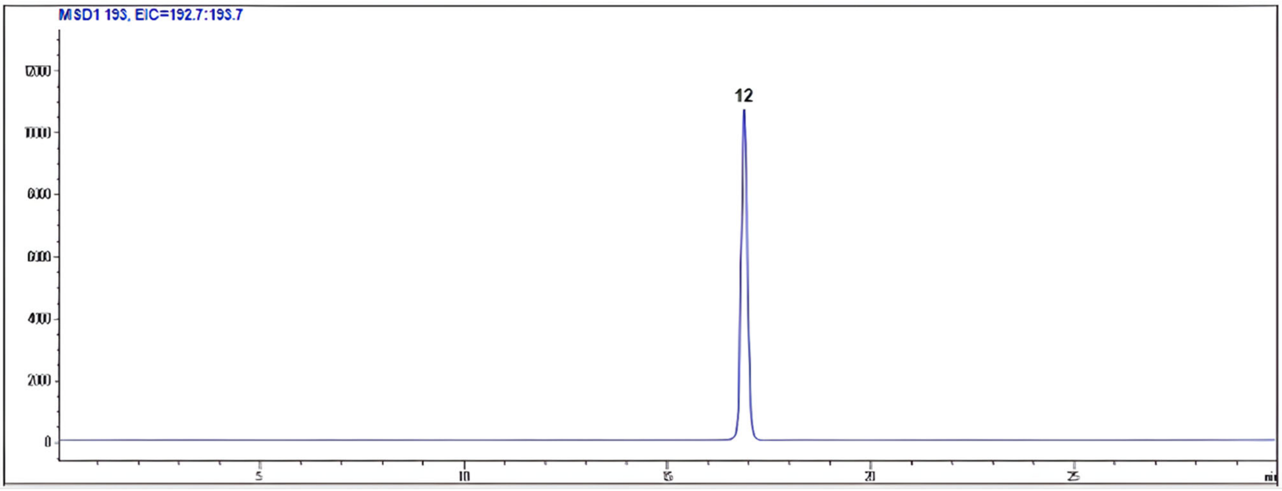
MSD1 193, EIC=192.7:193.7
12

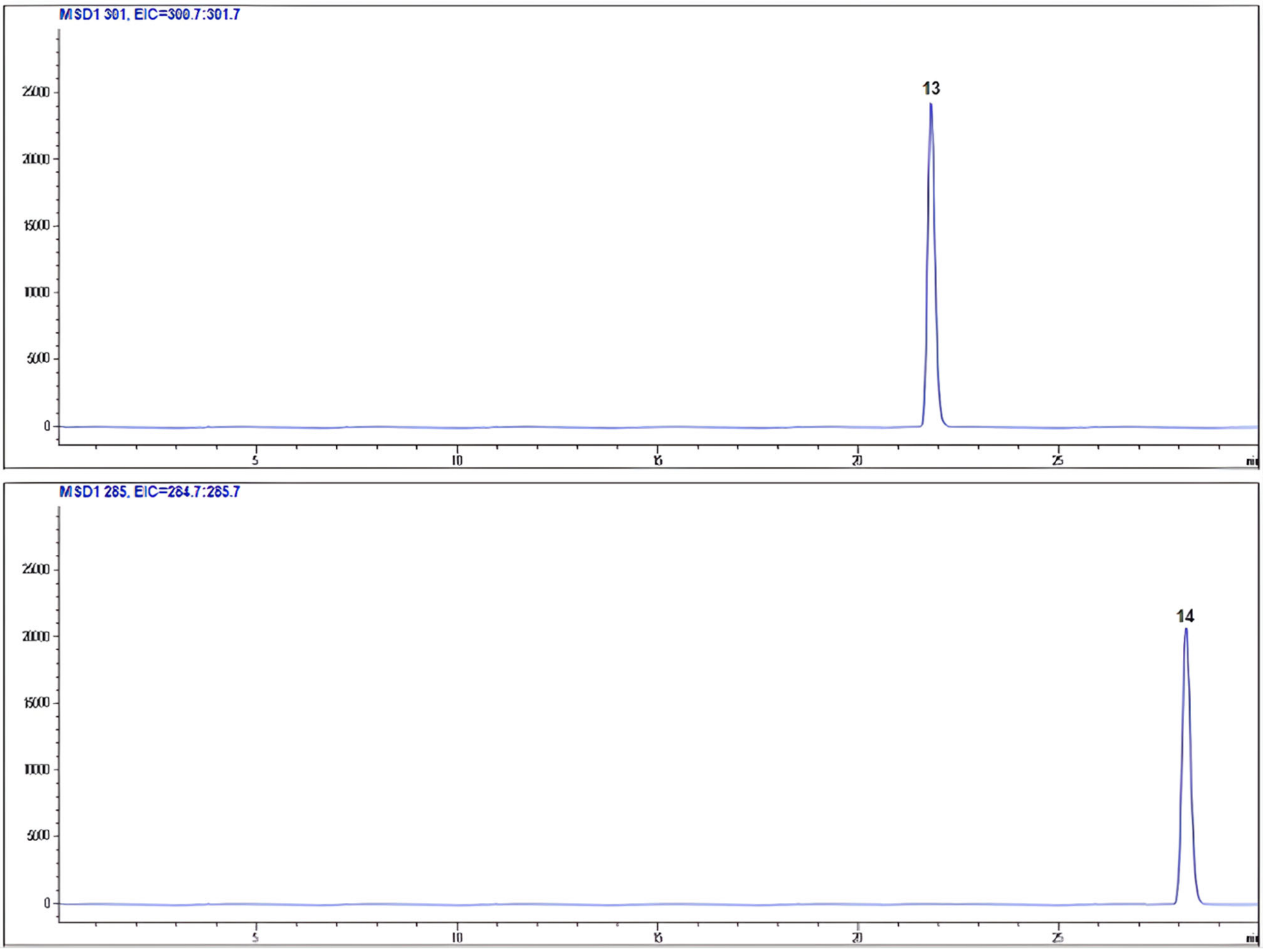


**Figure S2.** HPLC-ESI-MS/MS selected ion chromatograms (SIM) of the identified phenolic compounds from *F. varia* 70 % ethanol extract.
